# Relating increasing hantavirus incidences to the changing climate: the mast connection

**DOI:** 10.1186/1476-072X-8-1

**Published:** 2009-01-16

**Authors:** Jan Clement, Jurgen Vercauteren, Willem W Verstraeten, Geneviève Ducoffre, José M Barrios, Anne-Mieke Vandamme, Piet Maes, Marc Van Ranst

**Affiliations:** 1 Hantavirus Reference Center, Laboratory of Clinical Virology, Department of Microbiology & Immunology, Rega Institute, Minderbroedersstraat 10, B3000 Leuven, Belgium; 2 M3-BIORES, Biosystems Department, Katholieke Universiteit Leuven, W. de Croylaan 34, B3001, Heverlee, Belgium; 3 Epidemiology, Scientific Institute for Public Health, Juliette Wytsmanstraat 14, B1050, Brussels, Belgium

## Abstract

**Background:**

Nephropathia epidemica (NE), an emerging rodent-borne viral disease, has become the most important cause of infectious acute renal failure in Belgium, with sharp increases in incidence occurring for more than a decade. Bank voles are the rodent reservoir of the responsible hantavirus and are known to display cyclic population peaks. We tried to relate these peaks to the cyclic NE outbreaks observed since 1993. Our hypothesis was that the ecological causal connection was the staple food source for voles, being seeds of deciduous broad-leaf trees, commonly called "mast". We also examined whether past temperature and precipitation preceding "mast years" were statistically linked to these NE outbreaks.

**Results:**

Since 1993, each NE peak is immediately preceded by a mast year, resulting in significantly higher NE case numbers during these peaks (Spearman R = -0.82; P = 0.034). NE peaks are significantly related to warmer autumns the year before (R = 0.51; P < 0.001), hotter summers two years before (R = 0.32; P < 0.001), but also to colder (R = -0.25; P < 0.01) and more moist summers (R = 0.39; P < 0.001) three years before. Summer correlations were even more pronounced, when only July was singled out as the most representative summer month.

**Conclusion:**

NE peaks in year 0 are induced by abundant mast formation in year-1, facilitating bank vole survival during winter, thus putting the local human population at risk from the spring onwards of year 0. This bank vole survival is further promoted by higher autumn temperatures in year-1, whereas mast formation itself is primed by higher summer temperatures in year-2. Both summer and autumn temperatures have been rising to significantly higher levels during recent years, explaining the virtually continuous epidemic state since 2005 of a zoonosis, considered rare until recently. Moreover, in 2007 a NE peak and an abundant mast formation occurred for the first time within the same year, thus forecasting yet another record NE incidence for 2008. We therefore predict that with the anticipated climate changes due to global warming, NE might become a highly endemic disease in Belgium and surrounding countries.

## Background

**Figure 1:**
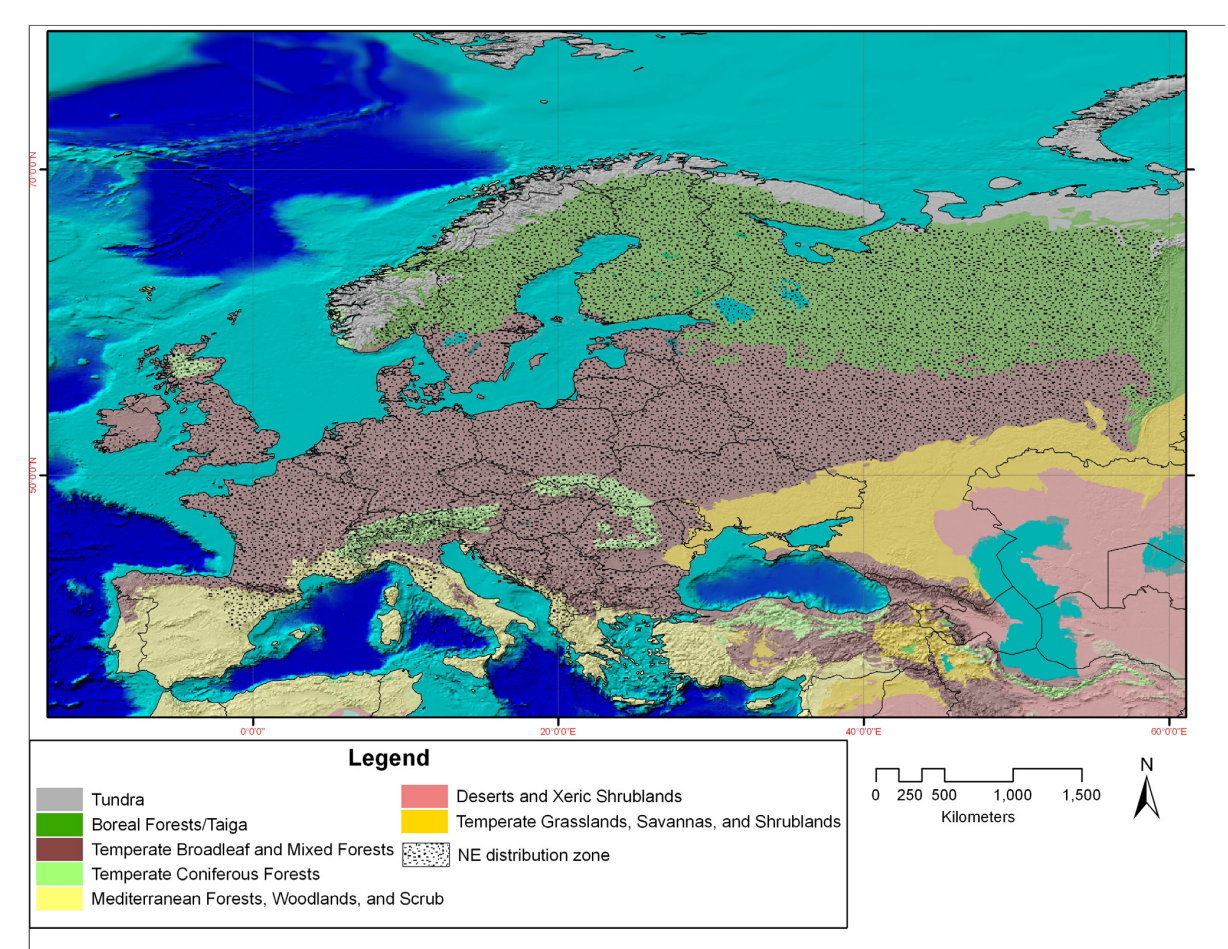
**Map of different biotopes and NE distribution in Europe and Western Russia**. Temperate broad-leaf forests and boreal forests are the preferred habitats of the *Puumala virus *rodent reservoir *Myodes glareolus*, and hence often concur with NE endemic zones, as indicated. However, some regions such as the U.K. have so far no known NE endemicity, despite having broad-leaf forests as an adequate biotope and a documented presence of *M. glareolus*. The vegetation map was derived from Olson, D. M, E. Dinerstein, E.D. Wikramanayake, N.D. Burgess, G.V.N. Powell, E.C. Underwood, J.A. D'amico, I. Itoua, H.E. Strand, J.C. Morrison, C.J. Loucks, T.F. Allnutt, T.H. Ricketts, Y. Kura, J.F. Lamoreux, W.W.Wettengel, P. Hedao, & K.R. Kassem.

Hantaviruses are worldwide emerging hemorrhagic fever viruses which are transmitted to humans via aerosolized excreta of chronically infected rodents, the main reservoir in nature. In Europe and Russia, the most important hantavirus is *Puumala virus *(PUUV), which is spread by a common wild rodent, the bank vole (*Myodes glareolus) *[1-3]. PUUV infection causes nephropathia epidemica (NE), a mild form of hemorrhagic fever with renal syndrome (HFRS), the general denomination of hantavirus disease in the rest of the Old World [1-3]. The preferred habitats of *Myodes glareolus *are temperate deciduous broad leaf forests that can be mixed with pine trees. This vole species is also common in boreal forests or taiga. Thus, NE is registered throughout Europe, except in northern Ireland, southern Spain and Portugal, and most of Italy and Greece, where these biotopes and consequently *Myodes glareolus *are lacking (Fig. 1)[1,3]. Hantavirus infections, and mainly NE, are a major problem in western-Russia, where each year morbidity and fatality rates reach far more important levels than in Europe [4]. For instance Bashkortostan, a republic in the European part of Russia with 4.1 million habitants, is one of the most endemic regions in the world for hantavirus infections: in 1997, more than 9,000 people contracted the disease, of which 34 cases were fatal [5].

Studying epidemiological features of an emerging disease is frequently hampered by the lack of sufficient follow-up in years. Belgium has a long-standing interest in NE, with the first serological evidence in Belgium reported in 1983 [6], a National Hantavirus Reference Centre operational since 1985, and since 1990 an official registry by the Scientific Institute of Public Health (IPH), Brussels. A sero-epidemiological study, mainly amongst healthy Belgian blood donors, was started in 1983 through to 1985, with 19,890 sera yielding a 1.35% PUUV seroprevalence, with a net predominance for the forested south of Belgium [7]. Thus, during an exceptionally long 25 year monitoring period (1983–2007), a total of more than 2,200 NE cases were registered, with a 0% fatality rate [8]. After the year 2000 however, there was a marked increase in frequency and magnitude of NE peaks which was impossible to ascribe to heightened medical awareness alone [9].

Global warming has been implicated or supposed to be responsible for an increase of some human and animal infections, in particular vector-borne infections. However, in the temperate regions of western Europe, hard data for an augmentation of such infections and their putative relation to global warming are so far scarce. The objective of our study was to examine the possible link of the observed NE increase in Belgium with (increased) temperature and precipitation. Some mechanisms that might explain important changes in driving forces, such as the local abundance of the rodent reservoir *Myodes glareolus *due to masting of the habitat vegetation, and a higher PUUV prevalence in this reservoir are discussed. This association could be highly relevant to other neighbouring countries like France, Germany, The Netherlands, and Luxembourg, where comparably higher NE incidences have recently been observed.

### Definition and hypothesis: the masting phenomenon

The staple food of bank voles consists of so-called "mast", or seeds of broad-leaf trees, mainly native oaks (*Quercus robur *and *Quercus petraea*) and common beech (*Fagus sylvatica*). A high mast production in autumn means a higher food supply for these rodents, which in turn means a higher survival rate and earlier breeding throughout winter, particularly if this winter is mild [10]. Mast abundance and perhaps also mild winter temperatures constitute important driving forces behind fluctuations in density of bank vole populations, which in temperate western Europe can reach up to 10 times the norm, leading to so-called "mice years", a vernacular term more used that the mammalogically more correct notion of "vole years" [3]. Tree seed production has already been linked to outbreaks of rodent populations in deciduous forests [11,12], but the cyclic character of this mechanism, and particularly its relevance to human pathology, has been less clearly studied. It is known, however, that a "mast year" can immediately precede sizable increases in bank vole populations during the winter of the same year, and in spring of the next (mast + 1) year [11-13]. Increases in bank vole densities have been frequently linked to increases in prevalence of PUUV infection in these voles, via direct or indirect transmission mechanisms [14-19]. Such a situation can result in the excretion of exceptionally high concentrations of infectious PUUV in nature, putting the local human population at risk [20]. Thus, according to our hypothesis, previously already formulated in 2002 and in 2005, peaks of NE in local human populations can ensue in the mast year + 1 [21]. Biomolecular evidence of this close human-rodent infectious relationship was seen in Germany during a major NE epidemic in 2007, by showing a close correlation between PUUV sequences obtained from both NE patients and bank voles from the same regions [22].

## Results

### NE infections and mast years

**Figure 2:**
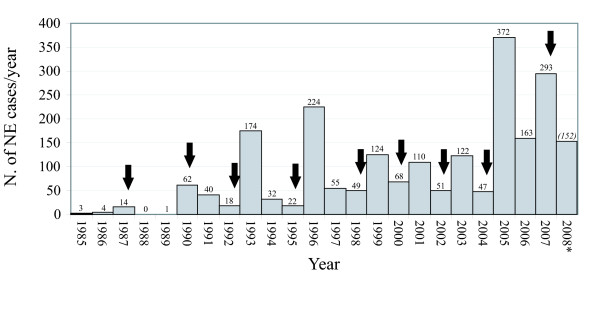
**Yearly numbers of NE cases in Belgium 1985-half 2008**. Serologically confirmed acute NE in Belgium, 1985-half 2008. For the current year 2008, marked with asterisk, only data of the first six months were available. Numbers above each column are the cases/year, the number between brackets above the 2008 column is only the half-yearly number. Mast years are indicated with black full arrows.

From 1985 to 2007, a total of 2,048 NE cases were registered, with increasing incidence throughout this 23-year observation period. With the provisional numbers of the first half of 2008 added, this total amounts now to 2,200. With these figures, NE by far exceeds the morbidity of leptospirosis, another mainly rodent-borne, but better known infectious nephropathy (only 77 cases for the period 2001–2007) [23]. Before 1999, only two NE peaks were recorded, both in the densely forested Belgian Ardennes, one in 1993 (174 cases) and one in 1996 (224 cases) (Fig. 2) [24,25]. The record year 2005 (372 cases) announced a quasi-continuous epidemic state for NE in Belgium. Of the total of 1,678 NE cases recorded in the here studied 12-year period (1996–2007), almost half (828 or 49.34%) have been documented in the last 3 years 2005–2007, equating to 276 cases/year in these recent years versus only 94 cases/year previously (P = 0.0031 two-sided t test with equal variances). This epidemic trend seems to be persisting for the current year 2008, with 152 cases already recorded during the first half year (weeks 1–24), compared to 150 cases during the first half of the previous record year 2005 (Table 1). 4-weekly NE cases, officially recorded by IPH from 1996 to 2007, and their relation to mast years, are given in Table 1.

**Table 1:** Seasonal distribution of the number of NE cases every 4 weeks in Belgium.

**Week**	**1995**	**1996**	**1997**	**1998**	**1999**	**2000**	**2001**	**2002**	**2003**	**2004**	**2005**	**2006**	**2007**	**2008**
**1 – 4**		13	5	2	8	5	4	7	7	5	17	4	16	28
**5 – 8**		17	2	3	6	9	7	4	4	0	22	9	14	28
**9 – 12**		11	3	2	4	7	9	0	9	3	14	16	12	16
**13 – 16**		11	4	1	7	2	10	1	4	2	19	18	27	23
**17 – 20**		15	5	6	14	4	19	3	21	1	33	13	35	20
**21 – 24**		23	1	4	9	2	7	3	9	2	45	18	29	37
**25 – 28**		19	3	5	18	8	15	5	16	5	63	14	21	
**29 – 32**		33	3	5	30	3	14	5	11	4	56	18	18	
**33 – 36**		23	1	6	18	6	11	4	9	4	37	8	26	
**37 – 40**		18	9	5	5	7	1	8	11	3	26	14	26	
**41 – 44**		11	9	3	1	5	7	3	10	7	12	9	20	
**45 – 48**		21	6	4	2	6	2	5	3	4	17	9	31	
**49 – 52**		9	4	3	2	4	4	3	8	6	11	13	23	
	*			*		*		*		*			*	

Mast years are indicated with *. Weeks 1–8 and 49–52 are winter periods. Weeks 9–20 are in spring. Weeks 21–36 are in summer. Weeks 37–48 are in autumn.

Mast years in Belgium occurred in 1987, 1990, 1992, 1995, 1998, 2000, 2002, 2004 and 2007, as indicated with full arrows in Table 1 and Fig. 2[13]. When these mast years are plotted against the yearly NE numbers on a 24-year time scale, it is striking that from 1993 onwards, all NE peaks were announced by a masting event the previous autumn (Fig 2). Moreover, numbers of NE in all these peak years are, at least since 1990, significantly higher than in the preceding mast years (Spearman R = -0.82, P = 0.034). During the first NE epidemic (1993), patients gave evidence of local abundance of bank voles after a mild '92-'93 winter, and plentiful acorns and beechnuts, suggesting that a masting event was already underway in 1992 for this earliest recorded hantavirus outbreak in Belgium and in France [3,13,24].

### Climate correlations

**Figure 3:**
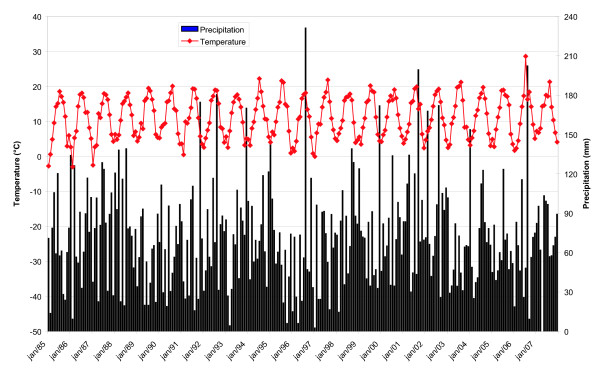
**Climate Graph Royal Meteorological Institute, Brussels, (Belgium) 1985–2007**. Monthly variations of mean temperature (red lines, in °C) and mean precipitation (black lines, in mm) of the current 1996–2007 study period, compared to the previous decade 1985–1995.

Temperature and precipitation profiles for the period 1985–2007 are given in Fig. 3. To check if the general impression of warmer and perhaps drier seasons is also valid for Belgium, we firstly compared mean daily temperatures in 1985–95 with the subsequent 12 years, i.e. 1996–2007 (Table 2). The year 1996 was chosen as a boundary point since it yielded the first of several other major NE peaks later on, and because 1996 was the starting year of 4-week NE epidemiological data recording, allowing statistical conclusions. We examined separately the two meteorological seasons considered crucial for tree fructification, i.e. spring and summer, versus autumn and winter, considered a crucial period for rodent survival. Finally, July was singled out as the most representative month for summer, since above-average temperatures and excess of sunshine in July one year prior to masting have been shown to stimulate flower bud formation [12].

**Table 2:** Comparison between climate variables in the current 1996–2007 study period and the previous decade.

	*1985–1995*	***1996–2007***	*P-value*
Average daily temperature in spring	10.3	10.9	0.0048*
Average daily temperature in summer	17.8	18.4	0.0001*
Average daily temperature in July	19.0	18.7	0.3033
Average daily temperature in autumn	11.0	11.7	0.0005*
Average daily temperature in winter	4.68°C	5.24°C	0.0048*
**Annual average daily temperature**	**10.7°C**	**11.4°C**	0.0000*

Annual average daily rainfall	2.30 mm	2.37 mm	0.5461
Average daily rainfall in summer	73.70 mm	81.30 mm	0.4722

Significance level of α = 0.05. Associations indicated in bold remain significant after sequential Bonferroni correction

The average temperature in the study period 1996–2007 (11.4°C) was statistically significantly higher than in the previous decade (10.7°C, P < 0.0001). This significant difference was also maintained when separate seasons (spring, summer, autumn or winter) were compared (Table 2). We did not find a significant difference however when only the month of July was examined. In fact, despite some record high July temperatures in recent years, July still appeared slightly colder (18.7°C) in the recent 1996–2007 period, as compared to the previous decade (19.0°C). Average precipitation throughout the year was almost similar (P = 0.546) in the two time periods. Summers precipitation during the last 12 years showed even slightly, but not significantly, higher levels than before. The impression of drier weather, particularly during recent hot summers, stems probably from the fact that rainy days were indeed less numerous [26].

Secondly, potential correlations were examined between the NE numbers as given in Table 1, i.e. registered every 4 weeks from 1996 through 2007, and climate parameters (mean daily temperature and precipitation) in the same period, and additionally the three preceding years, i.e. from 1993 to 2007. Matching was carried out for climatic data of summers three, two, and one year before the NE incidences (Year-3, Year-2, Year-1), springs of Year-1, and the same year (Year 0) as NE occurrence. Autumns and winters one year before NE occurrence were likewise examined (Table 3). The same calculations were performed with only July singled out as the most representative summer month [10], and April as the most representative spring month [27] (Table 4).

**Table 3:** Univariate correlations between the yearly cases of NE and seasonal climate variables three (Y-3), two (Y-2), one year(s) (Y-1) before, or during the same year (Y 0).

	*Spearman R*	*P*
**Summer temperature Y-3**	**-0.25**	**<0.01**
**Summer precipitation Y-3**	**0.39**	**<0.001**
**Summer temperature Y-2**	**0.32**	**<0.001**
Summer precipitation Y-2	-0.03	0.73
Summer temperature Y-1	0.16	0.05
Summer precipitation Y-1	0.18	0.02
**Spring temperature Y-1**	**-0.29**	**<0.001**
Spring precipitation Y-1	0.05	0.53
Spring temperature Y 0	-0.04	0.72
Spring precipitation Y 0	-0.11	0.31
Winter temperature Y-1	-0.13	0.11
Winter precipitation Y-1	-0.19	0.02
**Fall temperature Y-1**	**0.51**	**<0.001**
Fall precipitation Y-1	-0.14	0.07

Associations indicated in bold remain significant after sequential Bonferroni correction

**Table 4:** Univariate correlations between the yearly cases of NE and climate variables of single months.

	*Spearman R*	*P*
**July temperature Y-3**	**-0.37**	**<0.001**
**July precipitation Y-3**	**0.35**	**<0.001**
**July temperature Y-2**	**0.34**	**<0.001**
July precipitation Y-2	0.20	0.01
July temperature Y-1	0.21	0.01
July precipitation Y-1	0.02	0.84
April temperature Y-1	0.17	0.04
April precipitation Y-1	-0.09	0.25
**April temperature Y 0**	**0.27**	**0.01**
**April precipitation Y 0**	**-0.30**	**<0.01**

Months representative for summer (July) or spring (April), three (Y-3), two (Y-2), one year(s) (Y-1) before, or during the same year (Y 0).

Associations indicated in bold remain significant after sequential Bonferroni correction

## Discussion

### NE incidences and their relation to mast years

Before 1990, the low to nil annual NE incidence is almost certainly due to low medical awareness for this emerging infection. Starting from 1990, however, there are 3-year NE peaks, and from 1999 even 2-year peaks, which cannot be attributed to fluctuating degrees of awareness or better disease monitoring. The recent epidemic situation in the years 2005–2007 has been confirmed in neighbouring countries, such as Germany, where the average of ~220 cases/year was surpassed with 448 cases in 2005, and even more spectacularly in 2007 with a record number of 1,687 NE cases [22]. Although NE often remains a very localized "place disease", there is no reason to believe that NE mechanics should be fundamentally different in neighbouring countries (Germany, France, The Netherlands and Luxembourg) having the same biotope, i.e. consisting mainly of deciduous broad-leaf forests, the same documented presence of the rodent PUUV reservoir *M. glareolus*, and the same recent climate changes. In Nordic countries such as Norway, Sweden and Finland however, these mechanics are different, since boreal forests present little or no broad-leaf mast production, and since NE peaks seem linked rather to a predator-prey cycle [15,17,18]. Although we do not have recent rodent capture data to prove the rodent link, other studies have convincingly showed the direct or delayed relationship between increased local populations of PUUV-infected bank voles and NE peaks in Belgium [28,29] and in Sweden [15,30].

The question is then to identify the driving factors behind these fluctuations of bank vole densities, and why they seem more pronounced during recent years. As hypothesized in our 2002 and 2005 communications, increased broad-leaf tree seed production may give a satisfying answer (Fig. 2) [21]. The most pronounced NE peak in 2005 (372 cases) was preceded in 2004 by the most pronounced mast production ever recorded in Belgium, particularly for beechnuts [13]. The alternation of mast/non-mast years since 1998 is explained by the fact that a broad-leaf tree is physiologically unable to produce maximal mast in two consecutive years, even under optimal weather conditions. Nevertheless, occurrence of three confirmed mast years within a mere five year period (2000–2004) has never been observed before in Belgium, at least not for beech. Moreover, this record has been confirmed in several neighbouring countries, such as Germany [13].

The apparent absence of heavy masting in 2006, followed by another mast year 2007 is a particular case. Indeed, mast production at the end of the year 2004 had been so massive that beechnuts and acorns were left in profusion and lasting the majority of the next summer and autumn 2005. This way, we propose that the local vole population could have entered the subsequent 2005–2006 winter with an extra "left over" provision of conserved mast. High production of mast was observed in a 14-year-study in the Eastern USA, resulting in stores of acorns lasting throughout winter and well into the following year, instead of depletion by the month of January [31]. Moreover, early winter vole survival was promoted by a very mild autumn 2005, with a warm September (mean 17.1°C, norm 14.6°C), and an exceptionally mild October (mean 14.6°C, norm 10.4°C). The combination of a higher food supply with higher autumn temperatures might be reflected in the very elevated number of NE cases noted at the end of 2005 (total of autumn + December: 66), and the fairly high number in the next year 2006 (Table 1). Indeed, with 163 cases, this number of cases is the fifth highest in 12 years (Fig 2), but for once was not preceded by a heavy mast year, potentially for the reasons explained above.

### Climate influences on masting and NE incidence

The relationship between mast/NE peaks, and the recently higher frequency of both already suggests the influence of climate factors, particularly higher temperatures, as described by others [10-12,27,30]. A separate discussion of the four seasons is given below, as well as the influence of humidity and human exposure on NE incidence:

#### Spring period

Frost in April of the mast year (Year-1) was reported to reduce seed production [27]. Prolonged frost so late in the year is becoming increasingly rare however, and the record mast formation at the end of 2004 for instance was favoured by an abnormally mild April 2004 (mean 11.6°C). There is no straightforward explanation for the negative and significant correlation found for spring temperatures one year before NE occurrence, although this was not confirmed when April was singled out as a representative spring month (Tables 3 and 4). Flower induction of broad-leaf trees was observed to be stimulated by a warm and sunny spring, April in particular [27]. This cannot explain however the significant association found in this study between warmer and drier April months and NE numbers of the same year (Year 0), i.e. the year after mast formation (Table 4).

#### Summer period

Significant positive correlations were found between NE numbers and summer temperatures of year-2 (Table 3). Higher temperature of year-2 might stimulate bud formation in broad-leaf trees, acting as prerequisite for heavy masting at year-1, resulting in NE peaks in year 0. It is noticeable that the hottest summer in our 23 year (1985–2007) climate record was the year 2003, with mean temperatures for the three summer months June, July and August consecutively around or above 20°C (Fig. 3). Thus, the hottest summer (2003) ever recorded in Europe induced the largest mast production (autumn 2004) ever noted in Belgium [13], which in turn resulted in the highest NE peak (2005) observed so far. Furthermore, the latest mast year 2007 was preceded by another very hot summer (2006), with the mean temperature in July (23.0°C) the highest ever noted since the start in 1833 of the Royal Meteorological Institute (RMI) observations in Brussels [26]. (Fig. 3). In contrast again, mean temperatures in the summer therefore (2005) were normal to rather cool. Noticeably, the second hottest summer month ever, August 1997 (21.2°C), induced the 1998 mast year [26]. Summer precipitation of year-2 or year-1 seemed not to influence NE numbers at year 0. However, there was significant positive correlation with summer precipitation of year-3, and a weakly significant but negative correlation with the summer temperatures of year-3 (Table 3). As observed before [10], cold and moist summers appear to promote abundant masting two years later. Mean temperatures for July, as most representative summer month, showed an even stronger negative (R = -0.37) and highly significant correlation with NE incidences 3 years later, whereas presence or absence of other significant correlations for July were practically identical as for the whole summer studies (Table 4). It is noteworthy that in our comparative climate study July was the only summer month that appeared also slightly (but not significantly) colder than in the previous decade (Table 2).

#### Autumn period

The strongest positive correlation between temperatures and NE incidence was found for autumn the year before (Year-1) (R = 0.51; P < 0.001, see Table 3). This can explain the second highest (293 cases) NE peak in 2007, which has the particularity of combining for the first time a NE outbreak and a heavy masting within the same year (Fig. 2). We suppose there was only a moderate or "normal" mast production end 2006, with however a much higher survival of voles, most probably as a result of an exceptionally mild autumn and (see under) an exceptionally mild winter 2006–2007. Autumn 2006 had a mean temperature of 13.9°C (norm 10.4°C), a record so exceptional that, according to the statistics of RMI Brussels and in the hypothesis of a stable climate, such an event could only take place every 500 years [26]. Whereas October and November 2006 were, respectively, the second and fourth mildest ever, September 2006 was the warmest since the first RMI recordings in 1833 (mean 18.4°C, norm 14.6°C) [26]. In summary, the year 2007 witnessed a NE peak, induced by a record warm autumn (and winter), followed by pronounced masting, induced by a record hot summer, both in the previous year 2006.

Autumn climate factors cannot influence any more autumn mast formation of the same year, but can still greatly influence other food sources and the bank vole population itself. Greater availability of staple food during increased tree seed production in autumn may allow greater bank vole survival not only during the subsequent winter, but also already in the (late) autumn of the mast year itself, particularly if this is accompanied by milder temperatures, as observed during recent years. Under this hypothesis, the human population is already at risk from higher NE incidence during late autumn and early winter of a mast year itself (weeks 37 through 52 on Table 1). We see a confirmation of this hypothesis in the repetitive and increasing NE numbers in autumn and in December as recorded at the end of the mast years 2000, 2002, 2004 and particularly 2007 (Table 1). The only exception is 1998, probably due to the lower numbers recorded throughout the whole year. In contrast to the summer NE peaks considered, until recently, as typical for Western Europe, we are now observing "late autumn peaks" with totals outnumbering the summer totals (Table 1). Conversely, in all other (i.e. non-mast) years of this study, summer NE totals remain traditionally higher than totals of the end of the year. To our knowledge, a cyclic "autumn NE peak rule" for mast years has not been reported so far.

#### Winter period

Winter 2006–2007 with a mean temperature of 6.6°C (norm 3.1°C) was the mildest ever noted since 1833 [26]. In another Belgian study, lower winter temperatures appeared strongly linked to higher PUUV prevalence in bank voles, via a hypothetic "virus ecology" mechanism, being longer PUUV survival in the soil due to lower ambient temperatures [32]. Even with this putative mechanism, it is hard to explain the late winter and/or early spring NE peaks, as sometimes observed in our study, particularly in some February months. These peaks, for instance, were noted in February 2005 (22 cases) and 2008 (20 cases) (Table 1). Since these peaks were linked to rather an 'opposite' weather influence, i.e. warmer winters, we suppose that for these and other early year NE peaks, a "rodent host ecology" mechanism was operative, rather than a "virus ecology" mechanism. Moreover, no significant correlations were found between winter temperatures, nor precipitation, and subsequent NE numbers (Table 3). Significantly, harsh winters with mean monthly temperatures below 0°C, as still noted at the beginning of 1985 to 1987, were completely absent in the 1996–2007 study period (Fig. 3).

### The influence of humidity on NE incidence

Soil moisture is another important element in the "virus ecology" hypothesis, promoting local PUUV survival [32]. If in laboratory conditions high humidity has been proven crucial for PUUV survival [33], and if a humid environment might be considered beneficial for PUUV transmission between voles [14,17], it should be questioned however whether this factor has been influential in the recent rise of NE numbers in Belgium. High soil moisture was described as significantly associated with the number of NE cases in a 1994–2005 study period [32]. In our opinion, it seems unlikely that the two major mechanisms reported to be important for "virus ecology", i.e. lower temperatures and high soil moisture, could be evenly efficient in all seasons, causing human infections to the same degree throughout the year. Indeed, no correlations were found in our study between precipitation in all four seasons in years-2, year-1, and year 0 and NE numbers the following year (Table 3). Moreover, since most NE cases occur in summer, no summer of the record last three years 2005–2007 was particularly moist (normal or even below average, Fig 2), except for August 2006 (202.3 mm, norm 74.4 mm). As shown in Table 2, the mean summer precipitation of the period 1996–2007 was only slightly, but not significantly, higher compared with the 1985–1995 period. With recent seasons (particularly summer) and even whole years not being significantly wetter, but instead being significantly hotter than before, it is improbable that a climate-accentuated role of "virus ecology" could explain the recent epidemic trend of NE.

### The effect of human exposure on NE incidence

Even without taking into account the intervening mast phenomenon, neither "host ecology" nor "virus ecology" can fully explain exactly why summer NE peaks have been noted in each western European series, including ours (Table 1). There are no literature data to convincingly prove that each year the bank vole population reaches its maximum size during summer months, nor that its PUUV prevalence is invariably at its peak in summer, both factors of "host ecology". As for "virus ecology", it is unlikely that PUUV shed in the environment would attain its maximal infectivity during the hot and dry summer, and that this mechanism should work even more efficiently during the higher aestival temperatures of recent years.

The most straightforward explanation is human behaviour itself, with increased outdoor summer activity resulting in a closer contact with voles or their excreta. Human outdoor activity however is more difficult to assess than rodent abundance and PUUV prevalence. In western Europe, only two case-control studies are available so far to evaluate the risk of acquiring NE. Both case-control studies were carried out in the Ardennes after a local NE outbreak in Belgium [34] or in France [35]. Living or working in the forest bore a significant risk, but firewood- cutting and -handling in the forest emerged as the activity with by far the greatest risk in both studies. Bank voles prefer making their burrows under wood piles, probably for protection. The use of wood logs for heating and even for cooking is a local custom much more developed in the Ardennes than elsewhere, and is an example of a local, gender-independent risk behaviour, which is always important to assess, even in isolated cases of NE.

Whereas minor winter NE peaks are explained by a higher number of rodents making contact in human dwellings in search for food and shelter, the opposite (i.e. winter camping) can, exceptionally, also result in winter NE occurrence. In the region of Ulm, South-Germany, an explosive NE outbreak was described among American troops camping in vole-infested terrain near the Danube river for a winter exercise in January 1990 [36]. Other NE or HFRS cases mainly due to camping or rough sleeping have been reported in China [37], Greece [38], West-Germany [7], and in the French Pyrenean mountains [39]. Other authors agree that seasonal NE peaks are better explained by shifts in rodent and/or human behaviour, rather than rodent abundance or PUUV prevalence fluctuations [40].

## Conclusion

NE, a zoonosis scarcely known before 1990, has been increasing in incidence in Belgium with a cyclic pattern, to reach statistically higher and even epidemic proportions since 2005. NE is a rodent-borne infection, implying that it is at least partly climate-dependent. As predicted in our 2002–2005 hypothesis, a cyclic increase of broad-leaf tree seed formation, mainly beechnuts and acorns ("mast"), was confirmed as the causal link. Since 1993, each NE peak has been preceded by increased autumnal mast formation the year before, resulting in yearly NE numbers significantly higher than those during the mast years themselves. Mast years however show "late autumn peaks" instead of the classic NE summer peaks. A higher availability of staple food for the rodent reservoir *Myodes glareolus*, together with a higher autumn-winter survival of this rodent, explains the higher and cyclic NE occurrence in Belgium and in neighbouring countries, Germany in particular. Both mechanisms, mast formation and winter survival of voles, are temperature-dependent to such a degree that significant correlations appeared to exist, allowing reliable predictions of NE outbreaks based on climate parameters alone. In summary, outbreaks can be predicted by rather cold and moist summers 3 years before, hot summers 2 years before, and a warm autumn 1 year before NE occurrence. The month of July alone appeared an even better predictor than the whole summer as a season.

Finally, recent record high summer and autumn + winter temperatures are apparently further increasing both described mechanisms, with e.g. another very hot 2006 summer forecasting another record NE year in the making for 2008, again via major mast formation in 2007. The fact that the growing combined effect of hotter summer and autumn seasons is matched by a growing epidemic trend of NE in recent years, can be considered as an effect of global warming. To our knowledge, similar effects in other human infections in temperate Europe have rarely if ever been described so far. On the veterinary front however, the year 2006 witnessed in livestock the first outbreak ever recorded in northern Europe of bluetongue virus, with more than 2,000 confirmed cases. This was seen as a probable consequence of the hottest summer/autumn period since records began [41]. Its reappearance with greatly increased severity in May-June 2007 has been attributed in great part to the abnormally mild 2006–2007 winter [41,42].

If confirmed by other studies and in other countries, this predictive power of simple climate variables can be used by health authorities to adjust their prevention policies. Similar alarming forecasts for NE via higher temperatures have recently been made for 2008 in Sweden [43], and in Bashkortostan, Western Russia, where already in the first half of 2008, almost twice as many cases have been recorded as in the whole of 2007 [5]. Finally, changing climate factors might also influence other emerging infections in western Europe, such as Lyme borreliosis which has both a tick- and a rodent-transmission cycle. One of the principal rodent reservoirs for *Borrelia burgdorferi *is again *Myodes glareolus*, already studied here, along with some other local small rodents and insectivores. They will be the subject of another, partly prospective study.

## Methods

### Study subjects and laboratory methods

In the current study, only patients residing in Belgium with symptoms suggestive for NE were serologically examined. These symptoms consist mainly of fever with prostration, thrombocytopenia and varying degrees of acute renal failure [8]. From 1996 on, NE cases were recorded by IPH not only in years, but also in 4-week periods [9]. Individuals suspected of NE were screened using IFA and/or more frequently using ELISA, based on nucleoprotein of PUUV. A recent PUUV infection had to be confirmed by a positive IgM result by ELISA or IFA, together with a positive IgG titer.

### Climate data

Daily data of temperature (in degrees Celsius) and rainfall (in mm) from 1985 to 2007 (Fig 3.) were obtained from the Royal Meteorological Institute (RMI), situated in the centre of the country in Ukkel (Brussels). This station is considered to be representative for the Belgian territory, despite regional variations. These meteorological data can be matched with countrywide data of NE cases. The RMI data were used to calculate monthly means for statistical comparisons. Seasons were defined meteorologically as "spring" being March, April and May, "summer" as June, July and August, "autumn" as September, October and November, and "winter" as December, January and February. April was singled out as most representative spring month [27], and July as most representative summer month [10].

### Statistical methods

For comparison of climatologic data, the two-sample unequal variance t-test with a confidence interval of 95% was used.

For correlations between epidemiological NE data and temperature and precipitation, simple non-parametric univariate correlation analysis (Spearman) was applied using the free statistical software R (version 2.3.1). Significance on α was set on 0.05. To reduce the family-wise error rate, sequential Bonferroni correction was used.

## Competing interests

The authors declare that they have no competing interests.

## Authors' contributions

JC founded the National Hantavirus Reference Centre, gathered and seroconfirmed a majority of the here described NE cases, conceived the idea of the study, and wrote the manuscript. JV performed the statistics on NE-climate correlations. WWV delivered the climatic data, performed the statistics on these, and helped to draft the manuscript. GD delivered the official Belgian NE data from 1990 on, and the 4-week NE numbers from 1996 on. JMB delivered the colour map in Fig. 1. AV supervised the statistics, and critically revised the manuscript for important intellectual content. PM prepared the figures and the outlay, and helped to draft the manuscript. MVR initiated the design and coordination of the study, and critically revised the manuscript. All authors read and approved the final manuscript.

## Footnote

Presented in part as an invited lecture at the Int. Summer Conference of the Society for Applied Microbiology (SFAM) Pathogens in the Environment and Changing Ecosystems, Nottingham University, UK, 8-11 July, 2002. (SFAM News 2002; 3: 26-39), at the annual meeting of the Belgian Society for Internal Medicine, Brussels, Belgium, December 9-10th, 2005, abstract 42 [21], and at the 7th International Congress on Hantaviruses, Buenos Aires, Argentina, June 13-15th 2007 "Predicting hantavirus outbreaks and Lyme borreliosis peaks in Belgium – and Europe: of mast, mice and men", proceedings, abstract S1-O2, p. 21.
